# A Simple Neural Network for Collision Detection of Collaborative Robots

**DOI:** 10.3390/s21124235

**Published:** 2021-06-21

**Authors:** Michał Czubenko, Zdzisław Kowalczuk

**Affiliations:** 1 Department of Robotics and Decision Systems, Faculty of Electronics Telecommunications and Informatics, Gdańsk University of Technology, Narutowicza 11/12, 80-233 Gdańsk, Poland; kova@pg.edu.pl; 2 Intema Sp. z o. o., Siennicka 25a, 80-758 Gdańsk, Poland

**Keywords:** cooperating robots, force and tactile sensing, robot safety, neural network applications

## Abstract

Due to the epidemic threat, more and more companies decide to automate their production lines. Given the lack of adequate security or space, in most cases, such companies cannot use classic production robots. The solution to this problem is the use of collaborative robots (cobots). However, the required equipment (force sensors) or alternative methods of detecting a threat to humans are usually quite expensive. The article presents the practical aspect of collision detection with the use of a simple neural architecture. A virtual force and torque sensor, implemented as a neural network, may be useful in a team of collaborative robots. Four different approaches are compared in this article: auto-regressive (AR), recurrent neural network (RNN), convolutional long short-term memory (CNN-LSTM) and mixed convolutional LSTM network (MC-LSTM). These architectures are analyzed at different levels of input regression (motor current, position, speed, control velocity). This sensor was tested on the original CURA6 robot prototype (Cooperative Universal Robotic Assistant 6) by Intema. The test results indicate that the MC-LSTM architecture is the most effective with the regression level set at 12 samples (at 24 Hz). The mean absolute prediction error obtained by the MC-LSTM architecture was approximately 22 Nm. The conducted external test (72 different signals with collisions) shows that the presented architecture can be used as a collision detector. The MC-LSTM collision detection f1 score with the optimal threshold was 0.85. A well-developed virtual sensor based on such a network can be used to detect various types of collisions of cobot or other mobile or stationary systems operating on the basis of human-machine interaction.

## 1. Introduction

We live in times when life expectancy is significantly increasing. Rapidly developing technology and widespread access to medicines make life easier, healthier and longer. Moreover, society in developed countries is aging and fewer and fewer people are choosing to work on manual production lines. As a result, there is a huge shortage of (not just physical) employees on the European labor market. The job vacancy rate increased from 1.3% in 2013 to 2.3% in 2019 [1]. According to a Boston Consulting Group report, in Poland, in 2030, for example, another five million employees will be needed. Due to the great monotony that characterizes many such jobs, human-replacement production robots are the perfect solution. In 2018, the highest concentration of production robots took place in Singapore (831 robots per 10,000 employees), the Republic of Korea (774) and Germany (338) [2]. The European average is 114 robots per 10,000 employees and is higher than the American (99) and Asian (91) [2] rates. Still, there is constant demand for robotization of enterprises.

Very often, robotic stations eliminate manual production lines and contribute to the elimination of human presence in production areas. Robots are usually surrounded by various types of barriers, fences and even cages. From the very beginning, manipulators are designed to recreate human operations, such as pulling, pushing, pressing and lifting elements. At the same time, they use great strength and speed, which makes them dangerous to humans. This trend is now evolving, and the industry is increasingly using collaborative robots called cobots, which first appeared in the late 1990s [3]. They are characterized by lower load capacity, reduced speed and reduced impact force. ISO/TS 15066 states that the maximum speed of the cobot should not exceed 250 mm/s measured in the center of the tool, while the maximum impact force should be less than 65–210 N (depending on the body part). Thanks to such restrictions, they are also safer for people. However, they are not widely used enough. In 2017, only 11,000 of the 400,000 robotic units were cobots (2.7%) [2]. This ratio increased to 3.3% in 2018. In recent years, more robots that are friendly to humans (in the sense of interaction) have emerged. Currently, they can also be equipped with an emotional expression system (e.g., the Baxter robot) or a dedicated emotional system (e.g., xEmotion [4]).

In order to interact freely with people, cobots should meet a number of safety standards (especially: ISO 10218 and ISO/TS 15066) [5]. On the basis of these standards, certain steps important for the safety can be distinguished:

- collision (event) prediction;
- pre-collision strategies (collision avoidance);
- collision event;
- post-collision strategies (stopping the robot).

Detection of events requires the implementation of appropriate sensors and algorithms (usually expensive). The following solutions are used to predict a collision event: optical zone barriers, pressure mats and strips, safety keys, motion and distance sensors, and vision sensors [6,7]. The pre-collision strategies take into account what will happen when an intruder enters the robot space. The technical specification ISO/TS 15066 concerns the reduction of the robot’s speed in the case of locating an intruder in the working area and supervising the separation of the robot’s trajectory from the human trajectory. To detect a collision event, we mostly use torque sensors. Commonly they are used for any degree of freedom. Note that there is also a Fanuc approach with an external torque sensor located at the mounting point between the foundation and the robot, which perfectly measures the cumulative effect of the acting force moments. To detect collisions, we also use touch mats (Bosch APAS system) or other sensors, such as CLS (Compliant Limb Sensor) [8], touch sensors [9] or other types of virtual sensors [10]. The post-collision strategies dictate what to do after a collision—it is basically a safety-related stop. Note that both strategies can be interpreted as an unconscious reaction to a specific stimulus: distal in the case of prediction and proximal in the event of a direct collision [11].

To reduce the costs of the pre-collision approach, one can use RGBD (Red-Green-Blue-Depth cameras such as Intel^®^ RealSense™ [12]. In this application, deep neural networks (e.g., YOLO—You Look Only Once—a real-time object detection system) used to detect an intruder provide good results. One can obtain even better results using neural networks (e.g., OpenPose) for detecting and estimating poselets (a poselet—positioning or position distribution—is a specific description, characteristics or structure, related to the mechanics of the human figure based on pose markers—key marker points, such as points on the feet, ankles, knees, hips, arms, elbows, wrists, neck and head—that suitably define a human pose or gesture) [6,13].

### 1.1. Contribution

This article proposes a solution to the problem of robot collision detection using a virtual force sensor based on neural networks. The topic of collision detection by neural networks is discussed in the literature [14,15,16,17]. However, only in a few cases, deep neural networks are used.

This article compares four different neural architectures. Moreover, two important innovations can also be identified here. The first concerns the use of Mixed Convolutional—Long Short-Term Memory layers [18], which results in the highest performance among the architectures tested. The second one concerns the analysis of the regression level, i.e., the number of previous samples used to excite neural networks. Consequently, a wide range of regression levels (4 to 32 samples) will be tested for each neural network and the experiments will show that the best accuracy in predicting the load current can be achieved by using a moderate depth of the regression level.

According to our information, there is no report considering different levels of regression (second novelty) for predicting a robot load current signal through a neural network. There is also no mention of a neural network based on a mixed convolutional LSTM layer (MC-LSTM) in the wide literature (the first novelty). Our tests prove that such a solution (MC-LSTM) is the best neural architecture for the considered load current prediction modeling task.

It should be emphasized that the presented dataset comes from the real-world prototype of the CURA6 production robot. The collisions will be triggered by appropriate real human-robot interactions. As the robot is a hand-assembled prototype, it is very difficult to determine its actual parameters (such as the center of gravity of each joint) with high accuracy. Hence, there is a fundamental problem in applying the classical modeling approach. Therefore, before it will be possible to compare white-box and black-box models, in our article, we analyze the influence of the type of neural network architecture and the regression level for the input data in solving the load current prediction task.

### 1.2. The Structure

Section 2 provides a mathematical formulation of the problem and an overview of the available solutions. Next, Section 3 presents the proposed black box approach to modeling the behavior of the cooperative CURA6 (Cooperative Universal Robotic Assistant 6) robot, equipped with a manipulator with six degrees of freedom. In particular, the black box predicts motor currents using artificial neural networks (ANNs). Four different approaches are tested: AR (Auto-Regressive), RNN (Recurrent Neural Network), CNN-LSTM (Convolutional Neural Network—Long Short-Term Memory) and MC-LSTM (Mixed Convolutional—Long Short-Term Memory). Then, a system for detecting failures (robot collisions) based on predictions and measurements of motor currents is proposed. Note that our system uses the aggregate detection and isolation effect, while the threshold method is used in further identification. The results obtained are discussed in Section 4. The last Section 5 presents the conclusions.

## 2. Problem Formulation

**Figure 1:**
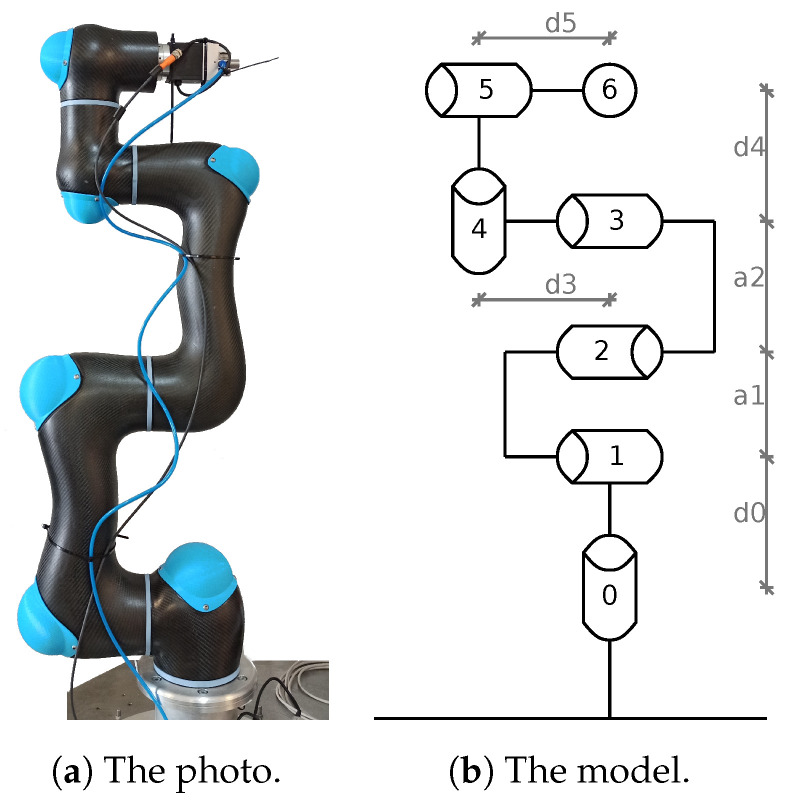
The CURA6 robot.

The main issue considered here is fast collision detection between a robot and a solid or moving body (obstacle or human). In the research reported here, the CURA6 industrial manipulator shown in Figure 1 with 6 DoF (Degrees of Freedom) developed by Intema was taken into account, the kinematic chain of which is characterized in Table 1 using the Denavit–Hartenberg notation. It is the original prototype of a new generation of cobots capable of moving a wide variety of objects weighing up to 5 kg over short distances (1200 mm).

In order to effectively handle such a manipulator, the inverse dynamics of the robot must first be determined. Although this task can be based on the analytical considerations [19], in most practical cases, it may turn out to be very difficult, and sometimes even impossible to carry out.

In the case of the CURA6 prototype, it is impossible to accurately assess the standard parameters required for the inverse dynamics problem (such as exact link mass, mass centers, and the exact model of the link itself). This difficulty is mainly due to the manual assembly of a single copy of this robot. In addition, when modeling the manipulator, it is difficult to take into account the weight of the connecting elements and cabling, not always routed along the link’s cross-section axis. The resulting error in estimating the model parameters significantly affects the problem of the manipulator’s inverse dynamics. Therefore, in this paper, we only cite research related to convenient modeling of robot behavior using neural networks.

Moreover, it is easier to obtain the parameters of such a dynamic model on the basis of an experiment with appropriate excitation and using an appropriate numerical algorithm (e.g., ABC—Artificial Bee Colony; GA—Genetic Algorithms, etc. [20,21]). It is worth noting that the visual identification method can also be used for this task [22]. There are a number of works that focus on determining the appropriate trajectories that trigger robot movements, whereby such a trajectory is a setpoint signal that stimulates system dynamics so that we obtain an over-determined system [23].

### 2.1. Dynamic Model of a Robot

The dynamic model of an industrial robot is given as:

(1) $$M\left(q\right)\ddot{q}+C\left(q,\dot{q}\right)\dot{q}+g\left(q\right)+\tau_{f}=\tau_{d}$$

where $q\left(t\right)=[q_{0}\left(t\right),q_{1}\left(t\right),\ldots ,q_{n-1}\left(t\right)]\in R^{n}$ encompasses the positions of each joint (n=6 in our robot), $\dot{q}\left(t\right)\in R^{n}$ and $\ddot{q}\left(t\right)\in R^{n}$ represent the velocity and acceleration vector, respectively. While $M\left(q\right)\in R^{n\times n}$ describes the robot inertia matrix, $C\left(q,\dot{q}\right)\in R^{n\times n}$ presents the effect of Coriolis and centrifugal force, $g\left(q\right)\in R^{n}$ describes the gravity force, $\tau_{f}\in R^{n}$ expresses friction forces (sum of viscous and Coulomb friction) and $\tau_{d}\in R^{n}$ is the joint torque vector [21,24].

With the Newton–Euler parameters, this model can be presented in the following affine (linear) form:

(2) $$\tau_{d}=\Phi \left(q,\dot{q},\ddot{q}\right)\theta$$

where $\Phi \in R^{n\times mn}$ is a Jacobian matrix and $\theta \in R^{mn}$ is a vector of manipulator dynamics parameters. As mentioned in the previous section, Equation (2) is commonly used to identify dynamics parameters using excitation trajectories based on Fourier series [20,21,25].

### 2.2. Collision Monitoring Methods

The virtual sensor concept is used in various approaches to detect manipulator collisions [26,27]. All such methods based on a virtual sensor, however, relate to the estimation of the external torque. Estimation of the external torque $\tau_{ext}$ can be made using an energy, velocity or torque observer. It can be determined directly by estimating the Φ matrix ($\hat{\Phi }$) or by using the inverse dynamics approach.

An excellent example of a force/torque sensor application for collision detection is given in [28]. The authors use filters of current signals (CF) to identify the type of collision along with statistically determined thresholds for the processed torque signal. While in the work of [29] a method based on low-pass and band-pass filtering was proposed.

There are also generalized momentum observer (GMO) methods that analyze the robot’s momentum change for collision detection [30,31]. Another method based on the momentum observer can be found in [32,33]. However, this is a model-based approach that is highly dependent on the quality of the dynamic robot model. For collision detection for robots with 6 DoF [26,30], the nonlinear noise observer (NDO) method can also be used.

A comparison of classical dynamic modeling (DM) with simple artificial neural network (ANN) modeling (and thresholding) is presented in [34]. There, the authors show that the respective threshold values applied to the torque error (in the range 1–2.5 Nm) lead to quite good performance when the torque calculated by the KUKA environment is used as a baseline. Their simple neural network (with 14 hidden neurons) is slightly more accurate, at the same time, it requires a well-prepared dataset. The article also presents a collision location method, but based on a larger neural network. In this case, the joint torque can be easily analyzed (in any context and without a model) for any abnormalities [35].

Another approach based on the use of neural networks for collision detection is presented in [17,36]. The authors present a simple neural network with 90 hidden neurons, which can estimate the robot’s torque for only one joint (with a fixed trajectory). Their neural network predicts torque with an average absolute error of about 0.09 Nm, and the accuracy of collision detection reaches almost 84%.

An interesting example of the use of artificial intelligence techniques can be found in [37], in which the authors use fuzzy residual evaluation (FRE) to detect faults.

In [32], the authors introduce a collision detection method based on the Yumi robot dynamics model to estimate the external force. The collision detector is implemented there as a single trigger based on the L2 norm of force estimation. On the other hand, [38] proposed a simple time-series approach (TSA) where the authors achieved 100% accuracy for two different trajectories (linear and arc).

Yet another approach is external proprioception based on an Integrated Measurement Unit (IMU) sensor [39]. Many other methods based on external complex physical or virtual sensors, such as cameras, 3d cameras, vibration analyzers and other [40,41,42], are also heading in this direction.

A short summary of selected features of the most popular methods in the context of collision detection is presented in Table 2.

### 2.3. Torque Estimation

Anticipating the possibility of a collision affecting robot segments, one should add the appropriate external moment to the robot dynamics Equation (2). The estimation of external torque can be written as the difference between the motor torque measured on the robot and its estimate:

(3) $${\hat{\tau }}_{\mathrm{ext}}\approx \tau_{d}-{\hat{\tau }}_{d}$$

The manipulator torque estimator ${\hat{\tau }}_{d}$ can be determined according to the robot dynamics model (calculated as a black box or using inverse dynamics). In practice, the above equation can be approximated as:

(4) $${\hat{\tau }}_{\mathrm{ext}}\approx K_{t}\cdot (I-\hat{I})$$

where I is the motor current, $\hat{I}$ represents the current estimate, and $K_{t}$ is a motor torque constant (which is different for different motors). In our case, the three main robot motors have $K_{t}=0.19$, while the others have $K_{t}=0.11$ (since CURA6 motors have a gear ratio of 1:100, the motor torque constant has to be multiplied by 100).

## 3. Black Box Model

Due to the high complexity of the problem of determining the robot dynamic parameters, we decided to model the robot using ANNs. Note that ANNs are commonly used in more advanced tasks [43]. A brief overview of the collision detection problem presented in the previous section indicates the legitimacy of using such a universal tool as ANN. In addition, it is well known that neural networks are effective in modeling time series (current waveforms of robot motors), although an appropriate set of data for ANN training is always needed.

### 3.1. Dataset—Experimental Setup

As training data, 15 slices of random movements of the robot were prepared, each containing about 10,000 samples (7 min of movement). Each motion trajectory was prepared by a trajectory solver that randomly selected a target point from the allowable work area. The speed of each joint was designed safely by the solver to be within 25% of the maximum speed of the motors.

**Figure 2:**
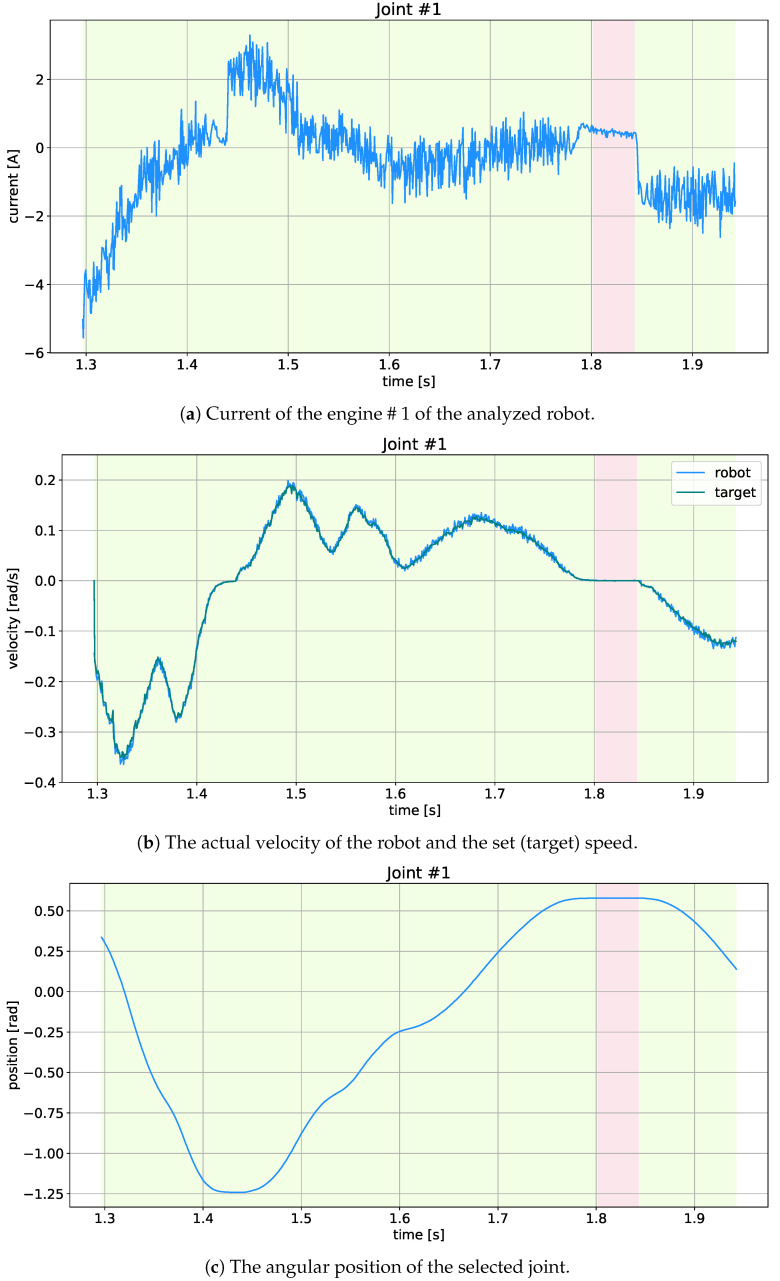
Example of 1000 samples (about 0.7 s) of measurement data for motor/joint # 1 (bright red stripe show the stop period while light green shows movement).

Three slices were collected without any load, and the others with the load from the following list {782, 1016, 1282, 2298, 2757, 4039} in grams, with two slices for each load (approximate load range was 500 g to 4000 g). These data were divided into learning (90%) and validation (10%) data. In addition, another set of test data (7 slices, one slice for each load) was prepared to determine hyper-parameters. Note that in our case the most important hyper-parameters are the order of regression and the ANN structure, while the less important are the batch size, number of epochs, and the optimizer and its parameters. Each slice contained four types of data: position (in rad), velocity (in rad/s), motor current (in A), input/controlling velocity (in rad/s), downloaded directly from the controllers of each degree of freedom (joint, motor) via CAN bus (Controller Area Network). The data sampling frequency was 24 Hz. Sample data for one slice (excluding the load) are presented in Figure 2.

Based on the collected data, approximately 30 learning datasets (each with a different regression order) were prepared. Their input dimensions (complexity) can be described as $batches\times 6\times 4\times regr^{{}^{\circ}}$ (number of learning samples × degrees of freedom × dimensions {position, velocity, motor current, input velocity} × regression order {number of time samples}), while the output has only two dimensions: batches×6 (the learning samples number × the motor current predictions number). The prepared datasets differ in the number of delayed samples depending on $regr^{{}^{\circ}}$ (the dataset for the 15th regression order has 162,905 samples, and for the 10th order—162,980).

Note that the concept of $regr^{{}^{\circ}}$ used in this article represents the regression order of prediction. Functionally, this parameter specifies the width of the time window or, equivalently, the number of previous samples used to predict the next motor current sample. In practice, the parameter $regr^{{}^{\circ}}$ can be translated into the input dimension of the system or the number of ANN layers.

As mentioned before, the CURA6 robot (Figure 1) performed the motions generated by the solver to a random point in the workspace. An obstacle (aluminum profile 40 × 40 mm, 1 m long) was placed on its trajectory to obtain data on the collision. This obstacle was held in the hand of the operator, so it can be considered static and somewhat susceptible to the impact force. The moment of the collision was recorded using a button. The collisions in the dataset were carried out at random points of all robot modules (all safety standards were met during data collection).

Note that all applied measurement data have not been filtered or pre-processed in any way. Moreover, an additional dataset with 390 collisions was prepared for external tests, which concerns 13 different loads and 7 different values of the maximum speed of the robot. All these datasets are publicly available at gitlab (since 2 June 2021) provided that this article is quoted at the same time.

### 3.2. ANN Models

Four models based on ANN were created:

(1) AR—Auto-Regressive model (perceptron);
(2) RNN—Recurrent Neural Network (ANN with two hidden recurrent layers);
(3) CNN-LSTM—ANN with time-distributed Convolutional Network (CNN) and Long-Short Term Memory (LSTM) layers;
(4) MC-LSTM—the ANN with a Mixed Convolutional-LSTM layer (based on [18]).

Each of the above ANN models has a different number of neurons, depending on $regr^{{}^{\circ}}$.

#### 3.2.1. AR Model (Perceptron)

**Figure 3:**
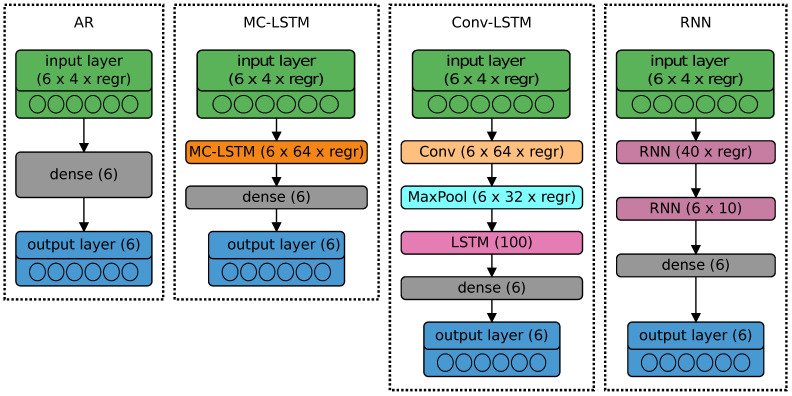
Structures of tested models predicting motor current ($\hat{I}$) on the output layer (6 neuron nodes), with a linear activation function.

The first neural network (Figure 3) imitating the AR model [44] consists only of the output layer with 6 neurons (current predictions, $\hat{I}$) densely connected to input nodes.

The simplest neuron equation can be written as:

(5) $$o=f\left(b+\sum_{k=1}^{N}w_{k}\cdot i_{k}\right)$$

where *o* is the output of the perceptron, $i_{k}$ is the *k*-th input of the neuron, *b* is a bias, $w_{k}$ is a weight, and *N* is the number of inputs.

When the activation function *f* is linear (i.e., ineffective because then the neural network loses its special non-linear properties), the output is assigned to the *t*-th time moment, and the input $i_{k}$ gets the time interpretation k=t−j; then, the perceptron can be described as a simple auto-regression model (AR):

(6) $$x_{t}=c+\sum_{j=1}^{N}\psi_{j}\cdot x_{t-j}$$

where *c* is the bias and $\psi_{j}$ represents the corresponding parameter (weight) of the AR model. Note that in the robot case all variables are vector quantities, thus, the whole model represents a vector auto-regression model.

#### 3.2.2. RNN Model

Recurrent neural networks (RNN) are characterized by good quality in time series forecasting (at the expense of longer learning time) [45]. A simple recurrent neuron can be described as

(7) $$o_{t}=f\left(b+u\cdot o_{t-1}+\sum_{k=1}^{N}w_{k}\cdot i_{k}\right)$$

where $o_{t}$ is the current output, $o_{t-1}$ represents the previous output (state) of the neuron, and *u* is its weight (which can be trained).

Such a model, initially constructed only using a single hidden layer (as the so-called vanilla model), turned out to be completely ineffective in the case of the CURA6 robot. Therefore, in this context, two recurrent layers were used, the first with the input complexity of $features\cdot 10\times regr^{{}^{\circ}}$ ($40\times regr^{{}^{\circ}}$), and the second with features×joints (4×6) dimensions. The output layer remained unchanged (consisting of 6 densely connected output neurons). For the sake of recursiveness, the RNN model created must remember several samples back (in additional regression layers). The applied model is presented in Figure 3.

#### 3.2.3. CNN-LSTM Model

Neural networks based on a convolutional layer (state-space aspect of systems modeling) and a long-short term memory memory layer (recurrent part with memory) are also great for predicting time series [46]. With the output layer similar to the above-mentioned ANN cases, the tested CNN-LSTM network was built on one convolutional layer (64 filters with Rectified Linear Unit, ReLU, activation function), a max pool layer (2 strides) and the LSTM layer (100 cells).

#### 3.2.4. MC-LSTM Model

As a fourth alternative, a neural network based on Mixed Convolution and LSTM layers was created [18]. In this case, in addition to 64 different convolutional filters, each LSTM cell also performs convolution on its four internal inputs (i.e., input, output, modulation and forgetting gates).

### 3.3. Learning Results

To properly select the hyperparameters, including the regression order, all considered models were trained according to prepared datasets for $regr^{{}^{\circ}}\in \langle 5;33\rangle$. The simple AR and RNN networks were matched using the Stochastic Gradient Descent algorithm, and the LSTM memory networks (CNN- and MC-LSTM models) using the Adam algorithm. In all cases, the learning rate was 0.001, and the mean square error (MSE) was used as the loss function. The batch size was 256, and the validation data accounted for 10% of the training set. The condition for completing the learning process was a minimum change in losses of $10^{-6}$ for four successive epochs.

**Figure 4:**
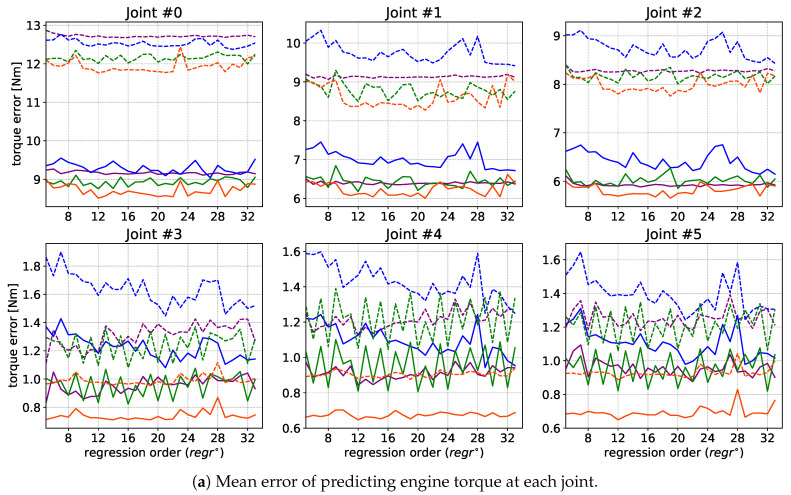

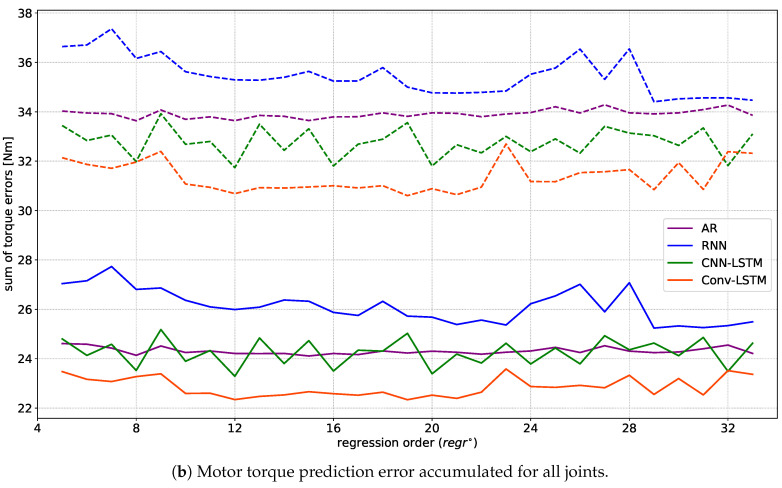
Experimental results for the mean absolute error—MAE (solid lines) and the root mean-square error—RMS (dashed lines) as a function of the regression order, where the descriptive legend is common.

**Figure 5:**
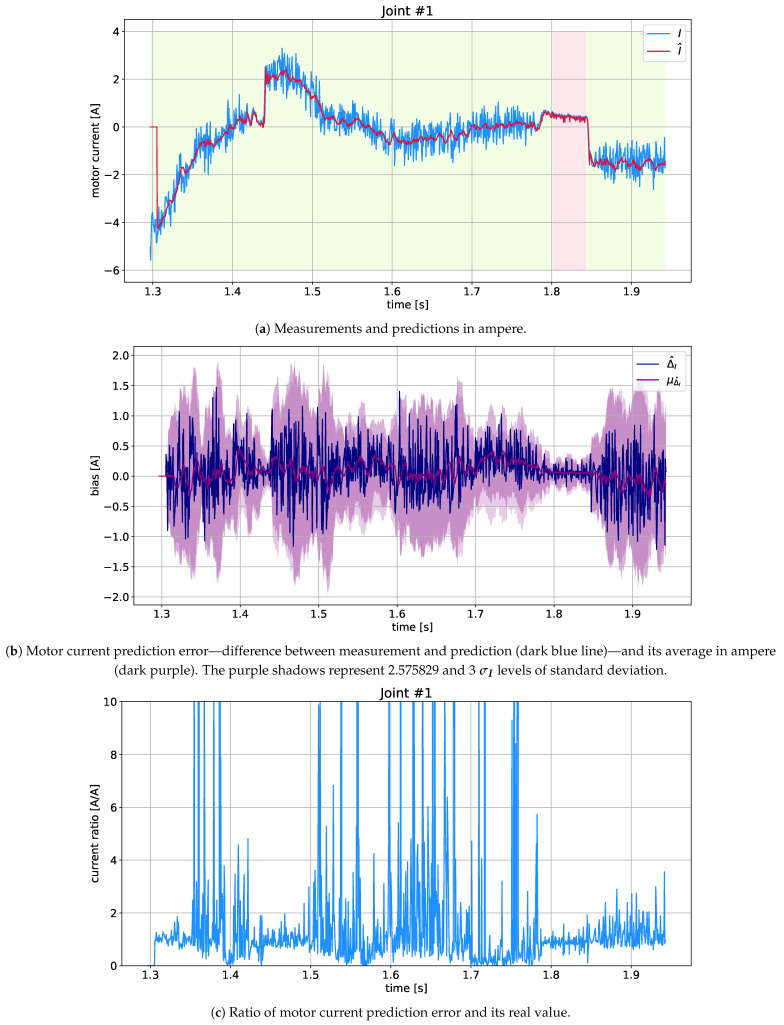
Current trajectories in ampere obtained using the MC-LSTM model for $regr^{{}^{\circ}}=12$ for example joint #1.

After training, each model was tested on an external test dataset (about 73,000). The target results for motor current predictions were calculated for each joint. To visualize the results, the Mean Absolute Error (MAE) was calculated for the adopted test dataset:

(8) $$MAE=\frac{1}{M}\cdot \left(\sum_{i=1}^{M}\left|I_{i}-{\hat{I}}_{i}\right|\right)$$

where $I_{i}$ is the measurement, ${\hat{I}}_{i}$ is the prediction for the relevant horizon (window), and *M* is the number of test samples in the test dataset. This result was scaled by a constant adequate for each motor to convert the motor current into motor torque. The mean torque differences for each joint are shown in Figure 4a, and their sum for all motors is given in Figure 4b (as solid lines). In addition, the Root Mean Square—RMS (dashed line) was calculated in both cases. One slice of validation data and the best model forecasts are shown in Figure 5. Note that Figure 5a presents the original current signal and its prediction, while Figure 5b shows the difference between them, its mean value and standard deviation. In turn, Figure 5c illustrates the ratio between the predicted and actual current signal, where all the visible peaks result from the almost zero value of the measured current.

It should be noted that with the dominance of the proposed approach, in fact, the differences between the analyzed methods presented in Figure 4 are very small. It should also be emphasized that the level of absolute values of the errors presented in this figure is not high. Apart from the fact that the data shown in Figure 4b have been accumulated over all joints, the torques and/or currents should be referred to the nominal values for the motors used. When we look at a single joint (e.g., # 1), the maximum MAE is below 7.5 Nm (approximately 7.4 Nm). This can be translated into a motor current value of 0.39 A (with $K_{t}=0.19$ and a gear ratio of 1:100). Meanwhile, the maximum current of this motor is about 48.8 A, and the current achieved in our tests was no more than 7 A. So, the average error when experimenting with the #1 joint was about 0.8%, and 7%—in the context of the maximum motor current range.

### 3.4. Summary

As can be seen in Figure 4a, there are three peculiarities: (*i*) the highest detectability, due to the highest current and torque values, is associated with motor number 1; (ii) the largest prediction error is related to motor #0 (responsible for rotation around the robot axis); (iii) the least effective regression orders are below 8, as well as between 22 and 30 (which is evident in the LSTM models). As for (ii): This phenomenon is caused by the nature of the robot’s main joint, whose motor is relatively evenly loaded over time and with low dynamics of changes. As for (iii): Although a single robot movement takes about 12 s (≈300 samples), high acceleration and deceleration phases occur within a range of less than 8 samples. Therefore, the robot motion characteristics should not be deduced from data obtained with a regression order less than 8. On the other hand, a clear decrease in the effectiveness of the MC- and CNN-LSTM models for $regr^{{}^{\circ}}\in \langle 22;30\rangle$ most likely results from the effect of the secondary time dependence associated with the long-term memorizing process occurring in these recurrent networks (roughly $2\times regr^{{}^{\circ}}$ in MC-LSTM and $3\times regr^{{}^{\circ}}$ in CNN-LSTM).

As can be estimated from the cumulative error (Figure 4b), the best filter is MC-LSTM with a suboptimal $regr^{{}^{\circ}}=12$ (RMS error about 31 and MAE about 22.5 in Nm). It is worth noting that this error is dominated by the effect of joint #0 (Figure 4a). As noted above (*i*), from the point of view of collision detection, the first joint (motor #1) is most important, which is the basic point of the robot lever arm and which is affected by all external torques.

It is interesting that our black-box approach gives similar accuracy (30 Nm of RMS error) as the classic white-box approach [21], without going into the complexity of the physical mathematical model. It should also be noted that analytical procedures are preferred by theorists, while ANN approaches can be widely practiced by industrial personnel.

In practice, according to the optimization carried out, we need the previous 12 samples to calculate the prediction. Note that such a single prediction takes about 0.20 ms on a standard computer (i5-8400, GTX 1070) using the CUDA library. However, on NVIDIA Jetson (target CURA6 controller) it takes a little longer—about 0.90 ms.

## 4. Collision Detection

Based on the ANN computations of Equation (4), impact detection occurs when the absolute value of the external torque estimate (${\hat{\tau }}_{\mathrm{ext}}$) exceeds a certain threshold ($\tau_{\mathrm{imp}}$):

(9) $$\left|{\hat{\tau }}_{\mathrm{ext}}\right|>\tau_{\mathrm{imp}}$$

According to Equations (4) and (9), an effective collision detector is:

(10) $$\left|I-\hat{I}\right|>I_{\mathrm{imp}}=\frac{\tau_{\mathrm{imp}}}{K_{t}}$$

The symptom of a collision with the robot can be sought in the phenomenon of a significant increase in motor current.

Motor current measurements are discrete, which is why discrete notation in time is appropriate, where *n* is the current moment. In this case, the current analysis horizon (sampling window) containing the last $regr^{{}^{\circ}}$ samples can be written as $\left[n-regr^{{}^{\circ}}:n-1\right]$.

### 4.1. Statistical Approach

With statistical argumentation and the assumption that the probability distribution of the motor current can be considered a normal distribution, the collision can be detected using the practical rule of three sigma [47]:

(11) $$P\left(collision\right)\approx P\left(\left|I\left[n\right]\right|>\gamma \cdot \sigma_{I}+\mu_{I}\right)$$

where $\sigma_{I}=\sigma \left(I\left[n-regr^{{}^{\circ}}:n-1\right]\right)$ is the standard deviation of the motor current, $\mu_{I}=\mu \left(I\left[n-regr^{{}^{\circ}}:n-1\right]\right)$ is the mean motor current, $I\left[n\right]$ is the actual motor current and $regr^{{}^{\circ}}$ is the regression order. Note that γ=2.575829 represents a 99% (probability) confidence level.

### 4.2. Prediction Approach

The left side of Equation (10) means the current prediction error (bias), which at time *n* can be shown as

(12) $${\hat{\Delta }}_{I}\left[n\right]\;≜\;\left|I\left[n\right]\;-\;\hat{I}\left[n\right]\right|$$

In a way similar to the statistical approach, $I_{\mathrm{imp}}$, the scaled threshold of Equation (10) can be computed as:

(13) $$I_{\mathrm{imp}}=\gamma \cdot \sigma_{{\hat{\Delta }}_{I}}+\mu_{{\hat{\Delta }}_{I}}$$

where $\sigma_{{\hat{\Delta }}_{I}}$ and $\mu_{{\hat{\Delta }}_{I}}$ represent the standard deviation and the mean of the motor current estimation error of the last $regr^{{}^{\circ}}$ samples. It should be clear that the threshold $I_{\mathrm{imp}}$ can be different for different types of performed movement (no movement, acceleration, uniform motion, braking). In dynamic cases (acceleration and braking), this threshold should be higher than in mild (semi-static) cases (as in uniform motion).

### 4.3. Results

Two inequality conditions are verified to detect collisions: (*j*) a purely statistical detector:

(14) $$\left|I\left[n\right]\;-\;\mu_{I}\right|>\gamma \cdot \sigma_{I}$$

and (jj) a prediction-based detector:

(15) $$\left|{\hat{\Delta }}_{I}\left[n\right]\;-\;\mu_{{\hat{\Delta }}_{I}}\right|>\gamma \cdot \sigma_{{\hat{\Delta }}_{I}}.$$

In addition, we will consider: (jjj) a reference, optimal detector based on the threshold analysis of the current prediction error given by Equations (10) and (12), and similar to Equation (15), with the threshold (2.0 [A]) precisely adapted to motor #1 and to the collision case under consideration:

(16) $$|{\hat{\Delta }}_{I}\left[n\right]\;-\;\mu_{{\hat{\Delta }}_{I}}|>2.0.$$

**Figure 6:**
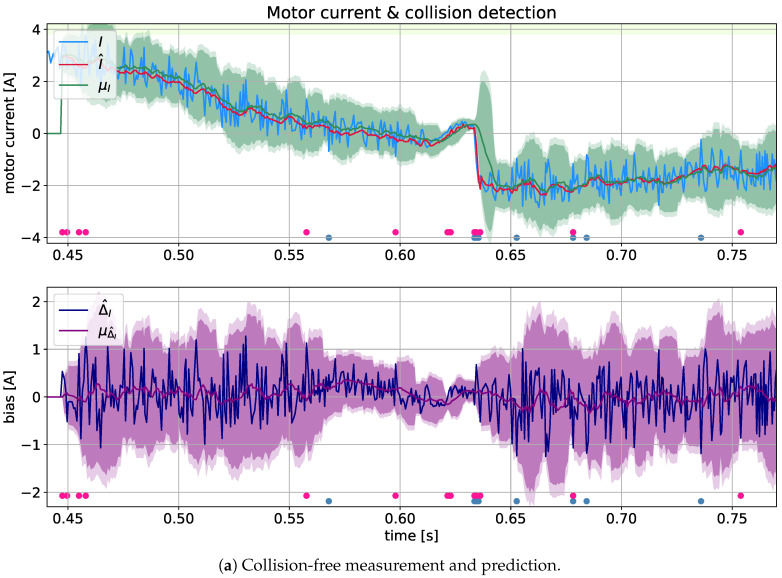

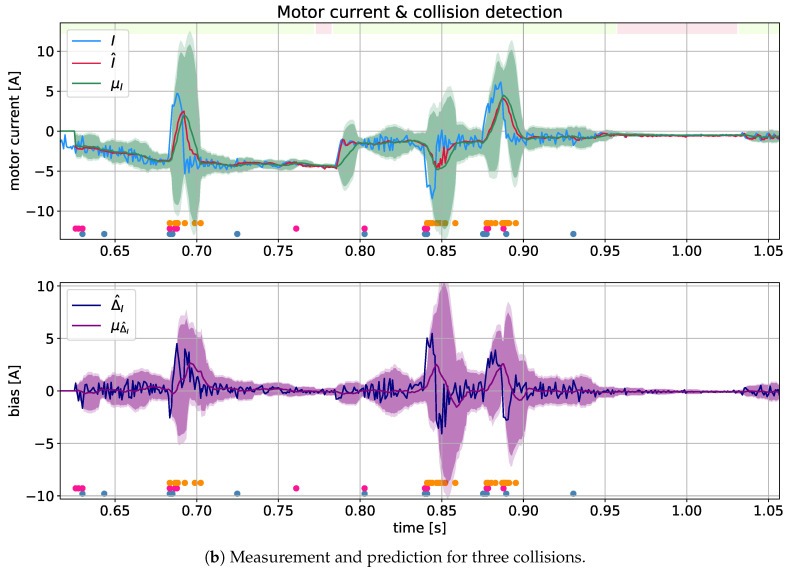
Diagnostic signals using the MC-LSTM(12) model for motor #1, including motor current *I* (blue line) and its prediction $\hat{I}$ (red line), mean motor current $\mu_{I}$ (green line along with green confidence area of width $\gamma \sigma_{I}$), and prediction bias, $I-\hat{I}$ (dark blue) relative to its mean (purple confidence area around zero of width $\gamma \sigma_{{\hat{\Delta }}_{I}}$). The statistical detection moments (*j*) are denoted by steel blue dots, while the prediction moments (jj) are marked as scarlet dots and the optimal threshold detector (jjj) is denoted by orange dots.

The effects of the collision detection analysis for motor #1 using the MC-LSTM model with $regr^{{}^{\circ}}=12$ are shown in Figure 6. The sub-figures (Figure 6a) show the signals without collisions. It should be noted that near the 280th sample, there is a sudden drop in the current signal caused by a dynamic change in the robot’s trajectory.

The sub-figures (Figure 6b) show three symptoms of collision events near samples 80, 250 and 290. It is worth noting that in the absence of a collision, there is a false signal increase near the 200th sample, which, however, is reduced by applying the deviation from the average.

In all upper graphs, the blue line shows the current (*I*) of motor #1, the red line is the prediction ($\hat{I}$), while the green line shows the mean motor current ($\mu_{I}$), and the green area depicts the confidence level $\gamma \cdot \sigma_{I}$. In the lower graphs, the deviation of the motor current from its predictions is shown by a blue line, and its mean is presented as the purple area corresponding to confidence $\gamma \cdot \sigma_{{\hat{\Delta }}_{I}}$.

The collision detection methods work on the basis of a one-step receding horizon. The statistical (*j*) detection moments are marked in Figure 6 as steel-blue dots, whereas the prediction (jj) detection times are marked as crimson dots. For reference (Figure 6b), the results of the optimal threshold detector (jjj) are marked as orange dots.

There are many false-positive events in both detectors (*j*) and (jj) (even in the collision-free case of Figure 6a). However, when one raises the confidence level (γ) (for example, to 3 or 5), true-negative detections will occur.

There are two other ways to reduce false-positive detections:

- logical multiplication (AND) on all three conditions;
- filtering several (2 or 3) detections in a row.

The use of logical multiplication resulted in effective collision detection. Filtering also gave good results, but at the cost of an additional detection delay of approximately 42 ms (one sample). Although, in principle, both methods are acceptable, in the case of fast-moving robots, additional filtration (depending on the sampling period) may be inconvenient.

### 4.4. Test on an External Dataset

Another test was performed with an external dataset (for 7 different speeds, where the maximum programmed speed of each joint was set to 10, 20, …, 60% of the maximum operating speed of the motors, and 13 different loads with the loads used in the external dataset were taken from the following list: {0, 401, 852, 1086, 1401, 1950, 2116, 2368, 2832, 3222, 3683, 4122, 4652}). The optimal threshold detector method (jjj) works well here: Out of 390 collisions, only 63 were not detected (and 52 false detections occurred). Note that the f1 score in this case was 0.85. Thus, the method is slightly better than the neural network mentioned in [36]. The method works independently for each joint, and the final collision verdict is based on any local collision (using the OR logic function).

**Figure 7:**
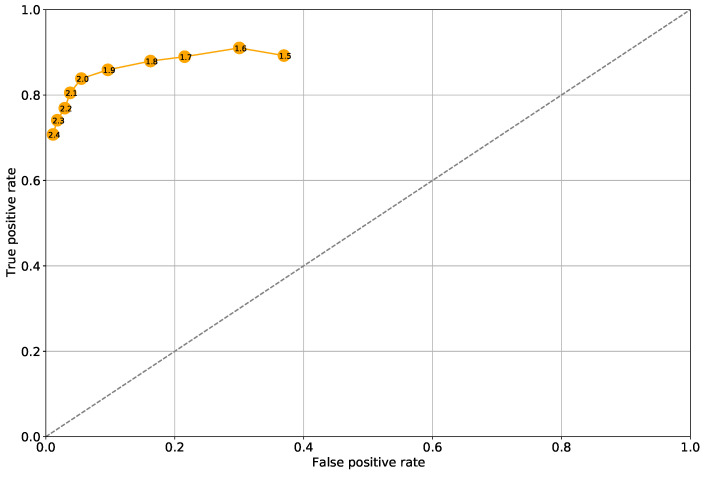
Receiver operation characteristic (ROC) for the MC-LSTM-12 model with thresholds (inscribed in tags) taken from the range (1, 2; 2, 1).

In order to clearly distinguish the situation of correct assessment, i.e., sensitivity or correctly positive decisions, from incorrect assessments, i.e., specificity or false positive decisions, we apply the so-called receiver operation characteristic (ROC) curve. With regard to the MC-LSTM-12 model with appropriate cut-off thresholds (inscribed in markers) taken from the interval (1.2; 2.1), such a ROC curve is shown in Figure 7. As can be seen in this figure, for many of the considered thresholds the detector is above the random guess (i.e., the diagonal: TPR = FPR), while the cut-off threshold of 2.0 [A] can be considered the optimum on the ROC curve.

The error matrix has also been computed for the external dataset as shown in Table 3. Note that the entire external dataset contains 78,000 samples in 78 different slices. Taking into account the established degree of regression, the first 12 samples from each slice were excluded for collision detection. Not every sample should be considered a possible collision detection. Formally, such a true negative value (TN) should be 76,622 (the Real TN rows in the table). However, in order to assume significant values, the mean slope of the motor current curve was determined for the samples assessed as FP. The TN data presented in the table using different speeds show no collision detection with a current curve slope greater than the mean for FP detections.

MC-LSTM has not been learned using such difficult and changing conditions as in the external dataset (in terms of speed and loads). As the training dataset was based on random paths, there is no requirement for a particular robot trajectory. However, during our testing, we discovered that the raw starting of the robot’s motors could be interpreted (mismatched) as a collision. We assume that fine-tuning and training on a larger dataset will improve the detection performance (ratio). It is also worth mentioning that the proposed method can be extended with the collision recognition function (with a dynamic or static obstacle). However, such a task will require deeper research and a different dataset.

### 4.5. Computing Resources and Timing

Taking into account the simple comparison of predicted and actual motor current values, the collision detection time can be limited even to the sampling period (42 ms for CURA6). Absolutely estimating, this time seems quite long compared to the results of other neural networks presented in [15], where the collision detection task performed with a MOD method takes 30 ms and 18.4 ms—in the case of CollisionNet (with a sampling period of approx. 0.25 ms). However, taking into account the relative computation time (in relation to the sampling period), our method outperforms the above-mentioned methods by about a hundred times. It should also be noted that the presented neural network (MC-LSTM12) requires 402,023 FLOPS (Floating-point Operations Per Second), mainly due to the large number of convolutions.

It is also worth noting that without taking into account the sampling time of the robots, the collision detection task takes about 0.20 ms on a standard computer (i5-8400, GTX 1070) using the CUDA library. The CURA6 controller (Jetson Xavier) needs a little more of this time to detect a collision—about 0.90 ms.

## 5. Conclusions

This article discusses collision detection using a virtual force sensor that processes information about motor current with the aid of an artificial neural network with four different architectures.

The conducted research proved (mean absolute error of prediction about 22.5 Nm) that ConvLSTM neurons [18] are the most suitable neural structure for modeling the dynamics of robots. It should be emphasized here that this robotic application of networks with ConvLSTM neurons is innovative. We do not know of any other work that implements a similar approach. The performed tests clearly showed that such a network exceeds classical neural networks in modeling inverse dynamics, and testing various regression horizons (stepping) shows that for the CURA6 prototype, a predictive network based on the previous 12 samples works best. It is obvious that this parameter (regression degree) is strongly correlated with both the sampling rate and the dynamics (speed of movement) of the robot.

The proposed method for determining the current of CURA6 robot motors has an accuracy similar to the accuracy of identifying a parameter matrix by means of evolutionary algorithms [21]. Considering the fact that the largest prediction error is related to the robot’s zero axis, and the error related to axis #1 (the most important from the viewpoint of detectability) is relatively small, it can be argued that the presented system allows for sufficiently effective collision detection.

The presented research proves that the monitoring of the difference between the measured and predicted currents allows for the detection of collisions. To reduce the number of false positives, in addition to appropriate conditioning (tuning) of the confidence levels, additional statistical processing can be used. The analysis conditions determined in this way allow for the detection of collisions for appropriately designed trajectories. In the case of very dynamic trajectories (multiple forward and backward jerk movements) the algorithm may erroneously detect collisions. The adaptive properties of the proposed detectors (statistical and predictive) in relation to the optimal detector, which was closely matched to the analyzed collision experiment, should be emphasized. It is also worth noting that optimal thresholding of the signal of the difference between the true and the predictive current allowed us to obtain the f1 score of 0.85.

Against the background of the results of the review of collision detection methods, MC-LSTM looks pretty good. The proposed approach only needs a well-prepared (and labeled) dataset, while analytical methods require detailed, precise robot parameters (which in the analyzed case of a manually assembled prototype could not be obtained). The use of MC-LSTM requires quite a lot of computing power, but only in the learning phase. It is also worth noting that the tool model of our robot was trained on a fairly simple dataset, and external tests were carried out under rather extreme conditions (taking into account the robot’s load and maximum speeds). From this point of view, it can be said that the results obtained exceeded our expectations.

Finally, it is worth mentioning that, in this article, we considered only the detection part, ignoring the objectives of isolation and identification included in the technical diagnosis or the FDI methodology [48]. In this context, isolation (and possibly identification) will be the subject of our future research. Therefore, in addition to extending our workshop to filtering motor current signals, we plan to develop a method of locating collisions (collision points) based on the measurement data of the motor current. It is also worth considering differentiating the type of obstacles into static and dynamic. Assuming the use of neural networks in such tasks (based on new datasets), it will be necessary to extend the neural architecture we have developed to include the analysis of the trajectory of load current signals at individual joints (apart from prediction of the main motor current). Of course, this approach should be related to the price and effectiveness of the “external” method of observing the environment.

## Figures and Tables

**Table 1:** DH parameters of CURA6.

*i*	$a_{i}$	$\alpha_{i}$	$d_{i}$	$\tau_{i}$	$\theta_{i}$
0	0	$\frac{\pi }{2}$	0.105	$\tau_{0}$	$\theta_{0}$
1	0.4	0	-	$\tau_{1}$	$\theta_{1}$
2	0.4	0	-	$\tau_{2}$	$\theta_{2}$
3	0	$\frac{\pi }{2}$	0.22	$\tau_{3}$	$\theta_{3}$
4	0	$-\frac{\pi }{2}$	0.2	$\tau_{4}$	$\theta_{4}$
5	0	0	0.14	$\tau_{5}$	$\theta_{5}$

**Table 2:** Comparison of collision detection methods, where CC is a computational complexity.

Method	Crucial Issues	Requirements	Limitations	CC
CF	filters design	current signal	different filers	low
GMO	friction disturbances	dynamics model	load	high
NDO	better sensitivity than GMO	friction estimation	load	medium
$\tau_{th}$	constant collision force	torque sensor	high price	low
DM		precise parameters		medium
$\hat{\Phi }$	precise parameters	adequate dataset		medium
Sensors		adequate *skin* sensor	sensor frequency	medium
TSA		torque signal	robot trajectory	medium
FRE	expert knowledge	expert rules		low
ANNs	learning	dummy dataset		high
MC-LSTM-12	learning	dummy dataset	lack of localization	high

**Table 3:** Confusion matrix when predicting collisions on the external dataset with the optimal method of collision detection at two sub-optimal current thresholds (1.6 and 2.0 [A]), where max *V* is the maximum programmed speed of each joint, th is the threshold, TP is true positive, FP is false positive, TN is true negative and FN is false negative (all values have been rounded to two decimal places).

max *V*	th	TP	FP	TN	FN	Recall	Precision	Specificity	Accuracy	f1-Score
10%	1.6	59	29	113	6	0.91	0.67	0.80	0.83	0.77
20%		60	39	136	5	0.92	0.61	0.78	0.82	0.73
30%		56	75	137	9	0.86	0.43	0.65	0.70	0.57
40%		62	64	159	3	0.95	0.49	0.71	0.77	0.65
50%		59	89	181	6	0.91	0.40	0.67	0.72	0.55
60%		59	87	167	6	0.91	0.40	0.66	0.71	0.56
Sum		355	383	893	35	0.91	0.48	0.70	0.75	0.63
Real TN		327	52	76,622	63	0.84	0.86	1.00	1.00	0.85
10%	2.0	52	3	113	13	0.80	0.95	0.97	0.91	0.87
20%		49	3	136	16	0.75	0.94	0.98	0.91	0.84
30%		52	9	137	13	0.80	0.85	0.94	0.90	0.83
40%		57	10	159	8	0.88	0.85	0.94	0.92	0.86
50%		58	15	181	7	0.89	0.79	0.92	0.92	0.84
60%		59	12	167	6	0.91	0.83	0.93	0.93	0.87
Sum		327	52	893	63	0.84	0.86	0.94	0.91	0.85
Real TN		355	383	76,622	35	0.91	0.48	1.00	0.99	0.63

## Data Availability

All data used for the experiments are publicly available at: https://gitlab.com/intema-gdansk/cura6-dataset.
